# SUN anchors pollen WIP–WIT complexes at the vegetative nuclear envelope and is necessary for pollen tube targeting and fertility

**DOI:** 10.1093/jxb/erv425

**Published:** 2015-09-25

**Authors:** Xiao Zhou, Norman Reid Groves, Iris Meier

**Affiliations:** Department of Molecular Genetics, The Ohio State University, Columbus, OH 43210, USA

**Keywords:** *Arabidopsis*, KASH, nuclear envelope, nuclear migration, plant fertilization, pollen tube ovular guidance, pollen tube reception, SUN, vegetative nucleus.

## Abstract

LINC (linker of nucleoskeleton and cytoskeleton) complexes play an essential role in nuclear migration by connecting the nucleus to the cytoskeleton and/or motor proteins. Plant LINC complexes have recently been identified in *Arabidopsis thaliana,* with the inner nuclear membrane SUN and outer nuclear membrane WIP proteins comprising the first identified complex. A recent study identified a nuclear movement defect in *Arabidopsis* pollen vegetative nuclei linked to the outer nuclear envelope WIP and WIT proteins. However, the role that SUN proteins may play in pollen nuclear migration has yet to be addressed. To explore this question, a SUN2 lumenal domain that was targeted to the ER specifically in pollen was over-expressed. It is shown that the ER-targeted SUN2 lumenal domain was able to displace WIP and WIT proteins from the pollen vegetative nuclear envelope. Expression of this dominant-negative transgene led to impaired VN mobility, impaired pollen tube guidance, and defective pollen tube reception. The observed pollen defects are similar to phenotypes observed in a *wip1-1 wip2-1 wip3-1 wit1-1 wit2-1* mutant. It is also shown that these defects were dependent on the KASH-binding function of the SUN2 lumenal domain. These data support a model where LINC complexes formed by SUN, WIP, and WIT at the VNE are responsible for VN migration and suggest an important function of SUN, WIP, and WIT in pollen tube guidance and reception.

## Highlight

*Arabidopsis* nuclear envelope SUN proteins are involved in transporting the pollen vegetative nucleus down the pollen tube and in efficient pollen tube targeting and male fertility.

## Introduction

In angiosperms, sperm cells are delivered to ovules by pollen. The pollen vegetative nucleus (VN) and the sperm cells [SCs, or their progenitor, the generative cell (GC)], termed ‘male germ unit’ (MGU), are usually closely associated and migrate together inside pollen tubes (Dumas *et al.*, 1985; McCue *et al.*, 2011). During migration, the VN precedes (is closer to the growing pollen tube tip than) the GC/SC in many angiosperm species including *Arabidopsis thaliana* (Heslop-Harrison and Heslop-Harrison, 1989; Lalanne and Twell, 2002; McCue *et al.*, 2011). That the VN and GC/SC migrate as a MGU was proposed to be important for successful fertilization by allowing signal transduction between the VN and SC (Dumas *et al.*, 1985) or by ensuring efficient and simultaneous SC delivery (Russell and Cass, 1981). However, the molecular mechanism for MGU migration has only recently been addressed in *Arabidopsis*, where VN movement is mediated by WPP domain-interacting proteins (WIPs) and WPP domain-interacting tail anchored proteins (WITs). The *Arabidopsis* genome encodes three genes for WIPs (*WIP1, WIP2*, and *WIP3*) and two genes for WITs (*WIT1* and *WIT2*) (Xu *et al.*, 2007; Zhao *et al.*, 2008). An *Arabidopsis wip1-1 wip2-1 wip3-1* triple null mutant (here abbreviated as *wip123*) and a *wit1-1 wit2-1* double null mutant (here abbreviated as *wit12*) shows a reversed VN-SC order and frequent loss of the VN during pollen tube growth, suggesting that the VN loses its locomotion and is transported forward by the SCs which still migrate towards the growing pollen tube tip. In addition, *wip123* and *wit12* exhibit significantly reduced male fertility, resulting from frequent failure of pollen tube reception, exemplified by overgrown pollen tubes inside ovules and polytubey (Zhou and Meier, 2014).

WIPs are outer nuclear membrane Klarsicht/ANC-1/Syne-1 Homology (KASH) proteins. KASH proteins interact with inner nuclear membrane Sad1/UNC-84 (SUN) proteins through the SUN–KASH domain interaction in the nuclear envelope (NE) lumen, forming linkers of the nucleoskeleton and the cytoskeleton (LINC) at the NE (Razafsky and Hodzic, 2009; Starr and Fridolfsson, 2010; Kim *et al.*, 2015). In opisthokonts, LINC complexes play an essential role in nuclear migration by connecting the nucleus to the cytoskeleton and/or motor proteins (Starr and Fridolfsson, 2010; Gundersen and Worman, 2013; Razafsky *et al.*, 2014). In *Arabidopsis thaliana*, SUN1 and SUN2 interact with WIP1, WIP2, and WIP3, forming NE bridges that anchor WIT1 and WIT2 to the outer nuclear membrane (Zhou *et al.*, 2015*a*). In roots and leaves, the SUN–WIP–WIT complexes recruit myosin XI-i to the NE, which mediates nuclear elongation and movement (Oda and Fukuda, 2011; Tamura *et al.*, 2013; Zhou *et al*., 2012, 2015*b*). Myosin XI-i is dispensable for pollen VN migration (Tamura *et al.*, 2013; Zhou and Meier, 2014), but whether SUN1 and SUN2 are involved in this process is unknown.

In this study, evidence is provided that SUN proteins are involved in VN migration during pollen tube growth. Due to the essential function of SUN proteins in meiotic chromosome movement, synapsis, and recombination, a *sun1 sun2* double null mutant has severe pollen developmental defects (Duroc *et al.*, 2014; Murphy *et al.*, 2014; Varas *et al.*, 2015), making it undesirable for this study. Here, the SUN2 lumenal domain was expressed under the post-meiotic, pollen-specific Lat52 promoter (Lat52pro) and targeted to the ER, thereby displacing WIPs and WITs (and potentially other known or unknown KASH proteins) from the pollen vegetative nuclear envelope (VNE). It is shown that this largely recapitulates the WIP and WIT depletion phenotypes, specifically when compared with a severe *wip1-1 wip2-1 wip3-1 wit1-1 wit2-1* (here abbreviated as *wifi*) mutant, but that a stronger effect on pollen tube guidance than reported before is caused by this approach. Together, the data presented here make SUN proteins most likely to be the inner NE players in pollen nuclear movement, thus suggesting that the WIP and WIT functions are indeed orchestrated in the context of a plant LINC complex.

## Materials and methods

### Plant materials

*Arabidopsis* plants were grown at 25 °C in soil under a 16/8h light/dark regime or on MS (Caisson laboratories) with 1% sucrose plates under constant light. The Columbia ecotype was used as the wild type (WT) unless indicated otherwise. *sun1-KO sun2-KD* was reported previously (Zhou *et al.*, 2012) and *WIP1pro::GFP-WIP1 wip123*, *WIT1pro::GFP-WIT1 wit12*, and *wifi* were also reported previously (Zhou and Meier, 2014). *sun2-1* was reported previously by Zhou *et al.* (2015*b*). *Lat52pro::GFP* WT was a gift from Dr R Keith Slotkin (McCue *et al.*, 2012).

### Constructs

Primers Lat52proinF and Lat52proIR were used to amplify the Lat52promoter from a *Lat52pro::GFP* construct (a gift from Dr R Keith Slotkin). Primers 2SAlbuminERF and 2SAlbminERinR were used to amplify the ERS (2SAlbminERF itself served as a template) by PCR. The above two PCR products were mixed and served as templates for overlapping PCR using Lat52proinF and 2SAlbminERinR as primers. The PCR product was then cloned into *Sac*I-and *Spe*I-digested pK7WGR2 by in-fusion (Clontech). After confirmation by sequencing, the pK7WGRERS52 vector was obtained. Primers 35SERSinF and 35SERSinR were used to amplify ERS from the pK7WGRERS52 vector and the PCR product was cloned in to *Spe*I-digested pK7WGF2 by in-fusion (Clontech). After confirmation by sequencing, the pK7WGFERS2 vector was obtained. All primer sequences are listed in Supplementary Table S2 at *JXB* online.

SUN2Lm was amplified by PCR from a SUN2 pENTR D/TOPO clone using SUN2LmF and SUN2LmR as primers. SUN2Lm PCR produce was cloned into pENTR D/TOPO (Life Technologies). After confirmation by sequencing, SUN2Lm was moved to pK7WGRERS52 and to pK7WGFERS2 by LR reactions (Life Technologies) to obtain *Lat52pro::ERS-RFP-SUN2Lm* and *Cauliflower Mosaic Virus 35S Promoter (35S*)-driven *ERS-GFP-SUN2Lm*, respectively. SUN2dMut was amplified by PCR from a SUN2dMut pENTR D/TOPO clone using SUN2LmF and SUN2LmR as primers. SUN2Lm PCR product was cloned into pENTR D/TOPO (Life Technologies). After confirmation by sequencing, SUN2dMutLm was moved to pK7WGRERS52 and to pK7WGFERS2 by LR reactions (Life Technologies) to obtain *Lat52pro::ERS-RFP-SUN2dMutLm* and Cauliflower *35S*-driven *ERS-GFP-SUN2dMutLm*, respectively.

### Generation of transgenic plants

Binary constructs were transformed to *Agrobacterium* strain ABI by triparental mating (Wise *et al.*, 2006). The ER-mCherry marker *Agrobacterium* strain (Nelson *et al.*, 2007) was obtained from the Arabidopsis Biological Resource Center. Transgenic *Arabidopsis* lines were obtained by the *Agrobacterium*-mediated floral dip method (Clough and Bent, 1998).

### Hoechst 33342 staining

For Hoechst 33342 staining, a solution containing 4% paraformaldehyde, 18% sucrose, and 4 µM Hoechst 33342 was used to stain pollen tubes for at least 20min. Pollen tubes were viewed under a Nikon C90i microscope. The UV-2E/C filter cube (Nikon) was used for imaging the Hoechst 33342-stained VN and SN. Images were taken by a Nikon DS-Qi1Mc digital camera.

### *In vitro* pollen germination and Alexander staining

Pollen grains from the stamens of fully opened flowers were germinated on a pollen germination medium containing 18% sucrose, 0.01% boric acid, 1mM CaCl_2_, 1mM Ca (NO_3_)_2_, 1mM MgSO_4_, and 0.5% agar. Several wild-type stigmas were placed adjacent to pollen grains to stimulate pollen germination (Qin *et al*., 2009, 2011). For the pollen competition assay, the pollen grains were directly germinated on stigmas. Alexander pollen staining was performed as described previously by Alexander (1969).

### Ovule imaging and pollen tube aniline blue staining

A magnifier was used to identify opening flowers with protruding unpollinated stigmas. These flowers were marked and the ovaries from these flowers were collected 3 d later. For imaging ovules, ovaries were dissected and ovules were mounted in 80mM sorbitol for microscopy (Leydon *et al.*, 2013). For aniline blue staining, ovaries were fixed in a solution containing acetic acid:ethanol (1:3 v/v) for 2h, washed in a 70%, 50%, 30%, 0% ethanol gradient for 10min each time, and softened in 8M NaOH overnight. Aniline blue solution containing 0.1% (w/v) aniline blue and 108mM K_3_PO_4_ (pH 11) was used to stain the softened ovaries overnight. Stained ovaries were dissected and ovules were imaged using a Nikon C90i microscope equipped with a UV-2A filter cube (Nikon). Images of pistils with stained pollen tubes were collected using a Nikon C90i confocal microscope. The aniline blue dye was excited by a 403nm laser and the emission above 470nm was collected as aniline blue signal.

### Co-immunoprecipitation experiments

*Nicotiana benthamiana* leaves were collected, ground to powder in liquid nitrogen, and Co-IP experiments were performed at 4 °C. One milliliter NP-40 buffer (50mM TRIS-HCl, pH 7.5, 150mM NaCl, 0.5% NP-40, 1mM EDTA, 3mM DTT, 1mM PMSF, and 1% protease inhibitor cocktail [Sigma-Aldrich]) was used to extract 500 μl of plant tissue. One-tenth of the protein extracts was used as the input sample and the rest were used for IP using protein A-sepharose beads (GE Healthcare) pre-coated with a rabbit anti-GFP antibody (catalogue number ab290, Abcam Cambridge). After washing three times in NP-40 buffer, the immunoprecipitates and the input samples were separated by 8% SDS-PAGE, transferred to PVDF membranes (Bio-Rad), and detected with a mouse anti-GFP (1:2000, catalogue number 632569, Clontech) or a mouse anti-Myc (1:1000, catalogue number M5546, Sigma-Aldrich) antibody. The input/IP ratio was 1/9.

## Results

### Single SUN null alleles cause no major fertility or VN movement defects

**Fig. 1.**
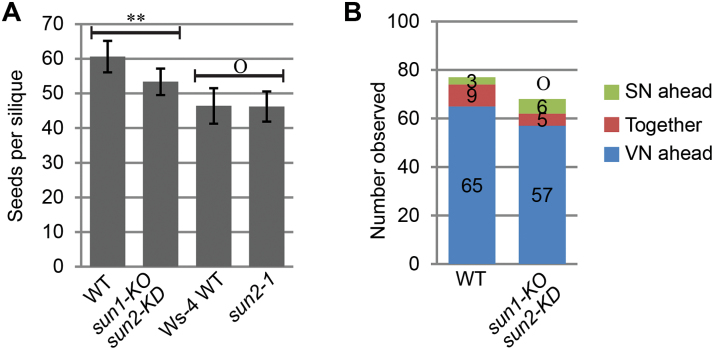
SUN1 and SUN2 function redundantly in seed production. (A) Seed count per silique of WT, *sun1-KO sun2-KD*, Ws-4 WT, and *sun2-1*. Double asterisks represent *P* <0.01, and ‘O’ represents *P* >0.05. Two-tailed *t* test was used and *n*=40. (B) *sun1-KO sun2-KD* has no VN migration defects. The nuclear order in the pollen tubes was examined 5h after pollen germination as described by Zhou and Meier (2014). Hoechst 33342 was used to stain DNA and the nuclei were imaged using fluorescent microscopy. Two-tailed Fisher’s exact test was used for statistical analysis and the numbers for each category are indicated on the graph. ‘O’ represents *P* >0.05, when compared with the WT.

Since the *wip123* and *wit12* null mutants have a severe reduction in seed set, seed production was analysed in the *sun1-knockout sun2-knockdown* (*sun1-KO sun2-KD*) mutant that recapitulates the *wip123* and *wit12* nuclear shape phenotypes in root hairs, trichomes, and root epidermal cells (Oda and Fukuda, 2011; Zhou *et al.*, 2012). Compared with the WT, *sun1-KO sun2-KD* has a small reduction of seeds per silique (12%) (Fig. 1A). This is far less severe than the 33% seed loss of the *wip123* mutant or the 50% seed loss of the *wit12* mutant (Zhou and Meier, 2014). To determine whether *sun1-KO sun2-KD* has impaired VN movement, its pollen nuclear order was examined 5h after pollen germination as described previously by Zhou and Meier (2014). Unlike the strong effect seen for *wip123* and *wit 12*, there is no apparent difference between *sun1-KO sun2-KD* and the WT (Fig. 1B) indicating that the VN of *sun1-KO sun2-KD* migrates normally in pollen tubes. This suggests either that SUN1 and SUN2 are not involved in the role of WIP and WIT in pollen nuclear migration or that the remaining amount of SUN2 in the *sun1-KO sun2-KD* is sufficient for this function. To dissect if *SUN2* is the main WIP and WIT anchor in pollen tubes, a *SUN2* null mutant in the Ws-4 ecotype, *sun2-1*, was acquired (Zhou *et al.*, 2015*b*). Figure 1A shows that *sun2-1* has no deficiency in seed production when compared with the Ws-4 WT. Together, these data suggest either that SUN1 and SUN2 are not involved in the role of WIP and WIT in pollen nuclear migration or that they act redundantly and—unlike in vegetative tissue—the remaining SUN2 in *sun1-KO sun2-KD* pollen is sufficient for this function.

### A mistargeted SUN2 lumenal domain depletes WIP1 and WIT1 from the pollen VNE

**Fig. 2.**
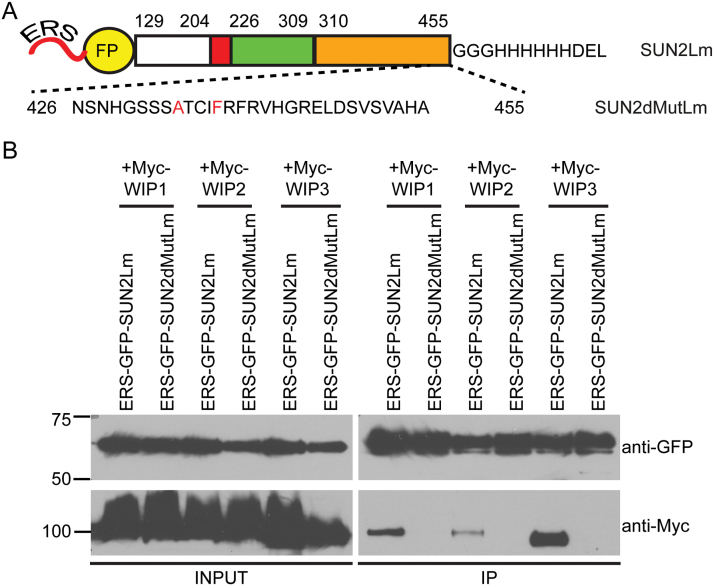
ERS-FP-SUN2Lm binds to WIP1, WIP2, and WIP3. (A) Domain organization of ERS-FP-SUN2Lm and ERS-FP-SUN2dMutLm. The lumenal fragment of SUN2 is drawn to scale. The numbers above each domain indicate the position of the first and the last amino acid of that domain. Key: white, unknown domain; red, coiled-coil domain; green, N-terminal part of the SUN domain; orange, C-terminal part of the SUN domain; ERS, ER signal peptide; FP, fluorescent protein. (B) Co-immunoprecipitation (IP) assay showing that ERS-GFP-SUN2Lm interacted with WIP1, WIP2, and WIP3, while ERS-GFP-SUN2dMutLm did not. Samples were co-immunoprecipitated with anti-GFP antibody. Input: IP=1:9

To address the requirement of SUN1/SUN2 for VN migration, a dominant-negative approach was therefore used as described previously by Crisp *et al.* (2006). The KASH-binding SUN domain of SUN2 was over-expressed in the ER lumen with the aim of outcompeting native SUN–KASH interactions at the NE. Specifically, a Golgi retrieval signal, HDEL, was fused to the C-terminus of the SUN2 lumenal domain (SUN2Lm). This construct was then N-terminally tagged with a fluorescent protein (FP) with an ER targeting signal (ERS), resulting in the ERS-FP-SUN2Lm construct (Fig. 2A). As a negative control, the NE lumenal domain of a SUN2 mutant carrying the H434A and Y438F point mutations (SUN2dMutLm) was used to make the ERS-FP-SUN2dMutLm construct (Fig. 2A). The H434A and Y438F point mutations impair the KASH binding ability of SUN2 (Zhou *et al.*, 2014).

Both ERS-GFP-SUN2Lm and ERS-GFP-SUN2dMutLm co-localized with an ER-mCherry marker in *Nicotiana benthamiana* leaves, confirming their presence at the ER (see Supplementary Fig. S1 at *JXB* online). Next, ERS-GFP-SUN2Lm or ERS-GFP-SUN2dMutLm was co-expressed with Myc-WIP1, Myc-WIP2, or Myc-WIP3 in *N. benthamiana* leaves to test for protein–protein interactions. An anti-GFP antibody was used to immunoprecipitate (IP) protein complexes from the cell extracts. As shown in Fig. 2B, Myc-WIP1, Myc-WIP2, and Myc-WIP3 interact with ERS-GFP-SUN2Lm, but not with ERS-GFP-SUN2dMutLm.

**Fig. 3.**
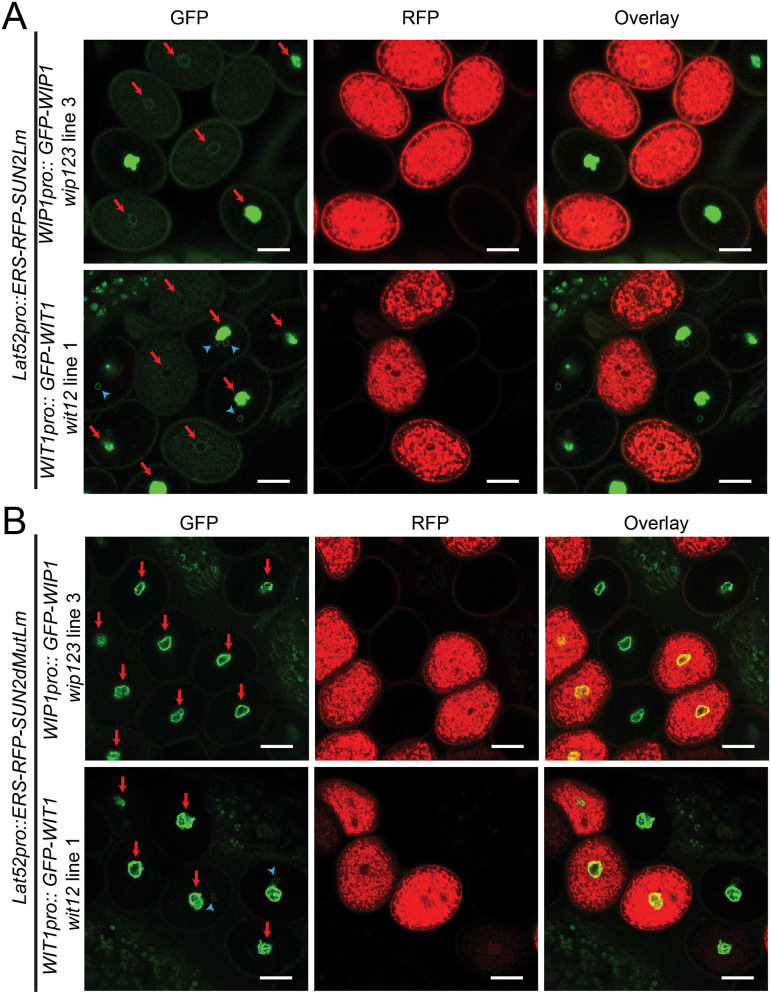
ERS-RFP-SUN2Lm delocalizes WIP1 and WIT1 from the pollen VNE. (A) *ERS-RFP-SUN2Lm* driven by *Lat52pro* was expressed in the pollen of *WIP1pro::GFP-WIP1 wip123* and *WIT1pro::GFP-WIT1 wit12*, respectively. The pollen grains of heterozygous plants were examined. GFP-WIP1 or GFP-WIT1 was delocalized from the VNE when ERS-RFP-SUN2Lm was expressed. The images were exposed such that the weak GFP signal at the VNE due to the displacement of GFP-WIT1 or GFP-WIP1 by ERS-RFP-SUN2Lm could be examined. Therefore, the VNE signal of GFP-WIT1 or GFP-WIP1 in pollen grains without ERS-RFP-SUN2Lm is overexposed. (See Supplementary Fig. S2 at *JXB* online for images with low exposure in the GFP channel showing the NE localization of GFP-WIP1 or GFP-WIT1.) (B) *ERS-RFP-SUN2dMutLm* driven by *Lat52pro* was expressed in the pollen of *WIP1pro::GFP-WIP1 wip123* or *WIT1pro::GFP-WIT1 wit12*. The pollen grains of heterozygous plants were examined. GFP-WIP1 or GFP-WIT1 was still localized at the VNE even when *ERS-RFP-SUN2dMutLm* was expressed. (A, B) The red arrows label the VNE and the blue arrowheads label the SC NE. Scale bars=10 μm.

To test whether expression of ERS-FP-SUN2Lm delocalizes WIPs and WITs from the VNE, an RFP version of this construct was generated and expressed in *Arabidopsis* under the post-meiotic, pollen-vegetative-cell-specific Lat52 promoter (Lat52pro) (Twell, 1992; Twell *et al.*, 1990). Lat52pro-driven ERS-RFP-SUN2Lm (*Lat52pro:ERS-RFP-SUN2Lm*) was transformed into *WIP1pro:GFP-WIP1*-rescued *wip123* line 3 and *WIT1pro:GFP-WIT1*-rescued *wit12* line 1, respectively, which have no pollen nuclear migration or seed production defects (Zhou and Meier, 2014). Figure 3A shows the resulting pollen grains segregating for ERS-RFP-SUN2Lm while expressing GFP-WIP1 or GFP-WIT1. The GFP fusion proteins were delocalized from the NE in pollen grains expressing ERS-RFP-SUN2Lm, but in pollen grains lacking ERS-RFP-SUN2Lm GFP-WIP1 and GFP-WIT1 strongly labelled the VNE (Fig. 3A; see Supplementary Fig. S2 at *JXB* online). The analogous experiment was carried out using ERS-RFP-SUN2dMutLm driven by Lat52pro (*Lat52pro:ERS-RFP-SUN2dMutLm*). As shown in Fig. 3B, ERS-RFP-SUN2dMutLm did not affect the localization of GFP-WIP1 or GFP-WIT1, although it was expressed at a similar level to ERS-RFP-SUN2Lm as suggested by the RFP intensity.

*Lat52pro::ERS-RFP-SUN2Lm* is likely to deplete other KASH proteins from the VNE. Additional KASH protein families have been identified, but only the SINE1/2 KASH family is also conserved across land plants (Zhou *et al.*, 2014). Other plant KASH families are present only in subgroups of plant species (Zhou *et al.*, 2014) and, therefore, they are less likely to play a role in the widely conserved process of pollen tube ovular guidance. Among *Arabidopsis* SINE1 and SINE2, SINE1 appears not to be expressed in pollen, but SINE2 is present at the SC NE and somewhat more weakly at the VNE (see Supplementary Fig. S3 at *JXB* online). However, despite the VNE localization of SINE2, *sine2* null mutants do not have a fertility defect (data not shown). Hence, it was reasoned that, in the context of known plant KASH proteins, the effect of *Lat52pro:ERS-RFP-SUN2Lm* is specific to WIP proteins.

### ERS-RFP-SUN2Lm causes severe seed loss in sun1-KO sun2-KD

If the remaining amount of SUN2 in *sun1-KO sun2-KD* is functioning in WIP and WIT VNE anchoring, then ERS-RFP-SUN2Lm in this background should compete with this interaction. Thus, *sun1-KO sun2-KD* was transformed with *Lat52pro::ERS-RFP-SUN2Lm* and *Lat52pro::ERS-RFP-SUN2dMutLm*, respectively, and homozygous transgenic lines were selected with similar expression levels.

**Fig. 4.**
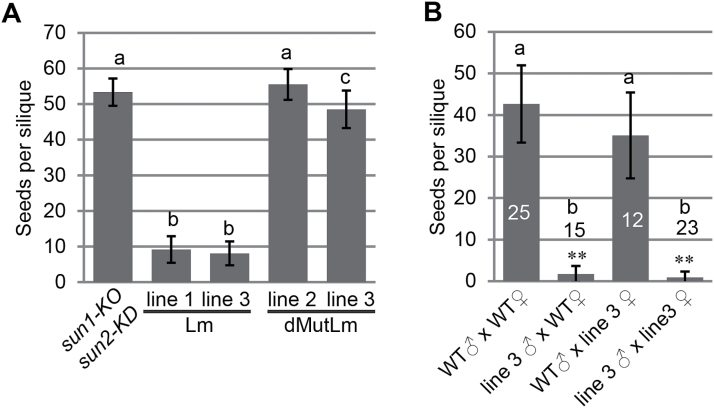
Expression of *Lat52pro::ERS-RFP-SUN2Lm* leads to severe male-fertility defects. (A) The number of seeds per silique between different lines showing that *Lat52pro::ERS-RFP-SUN2Lm* caused severe seed loss in *sun1-KO sun2-KD*. ‘Lm’ represents *Lat52pro::ERS-RFP-SUN2Lm sun1-KO sun2-KD*, and ‘dMutLm’ represents *Lat52pro::ERS-RFP-SUN2dMutLm sun1-KO sun2-KD*. For all samples, *n*=40. (B) Number of seeds per silique after reciprocal crosses between WT and *Lat52pro::ERS-RFP-SUN2Lm sun1-KO sun2-KD* line 3. The *n* of each sample is indicated in the histogram. (A, B) The error bars represent SD. One-way analysis of variance (α <0.01) followed by Tukey’s honest significant difference test (α <0.01) was used. Samples with the same letter (a, b, or c) show no pairwise statistically significant difference, and samples with different letters show statistically significant difference.

Figure 4 shows the seed set of two independent transgenic lines, each compared to *sun1-KO sun2-KD*. While *Lat52pro::ERS-RFP-SUN2Lm* lines had drastically reduced seed set, *Lat52pro::ERS-RFP-SUN2dMutLm* lines had seed set very similar to the *sun1-KO sun2-KD* background (Fig. 4A). Reciprocal crosses between *Lat52pro::ERS-RFP-SUN2Lm sun1-KO sun2-KD* line 3 and WT confirmed that the seed loss phenotype was derived from the male (Fig. 4B). It was then assessed whether expressing ERS-RFP-SUN2Lm or ERS-RFP-SUN2dMutLm in *sun1-KO sun2-KD* would affect pollen morphology or pollen tube growth. Supplementary Fig. S4 at *JXB* online shows that no visible difference was seen compared with the WT.

**Fig. 5.**
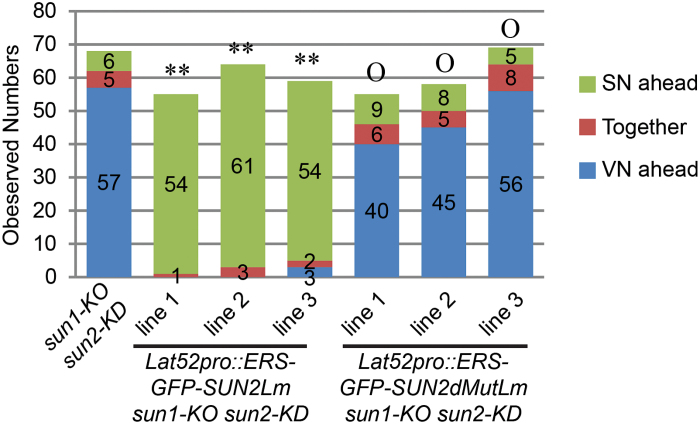
ERS-RFP-SUN2Lm, but not ERS-RFP-SUN2dMutLm, caused VN movement defects in *sun1-KO sun2-KD* pollen tubes. Nuclear order in pollen tubes was examined 5h after pollen germination, as described previously (Zhou and Meier, 2014). Hoechst 33342 was used to stain DNA and the nuclei were imaged using fluorescent microscopy. Double asterisks represent *P* <0.01, while ‘O’ represents *P* >0.05, when compared with the WT. Two-tailed Fisher’s exact test was used and numbers for each category are indicated in the figure.

To determine the position and order of VN and GC, pollen tubes were examined 5h after *in vitro* pollen germination and stained with Hoechst to visualize nuclei (see Materials and methods). Figure 5 shows that, in the majority of *Lat52pro::ERS-RFP-SUN2Lm sun1-KO sun2-KD* pollen tubes, the SN preceded the VN, very similar to the phenotype previously reported for *wip123*, *wit12*, and *wifi* (Zhou and Meier, 2014). By contrast, *Lat52pro::ERS-RFP-SUN2dMutLm sun1-KO sun2-KD* plants exhibited no change in pollen nuclear order when compared with untransformed *sun1-KO sun2-KD*.

### Pollen tubes expressing ERS-RFP-SUN2Lm have ovular guidance and reception defects

**Fig. 6.**
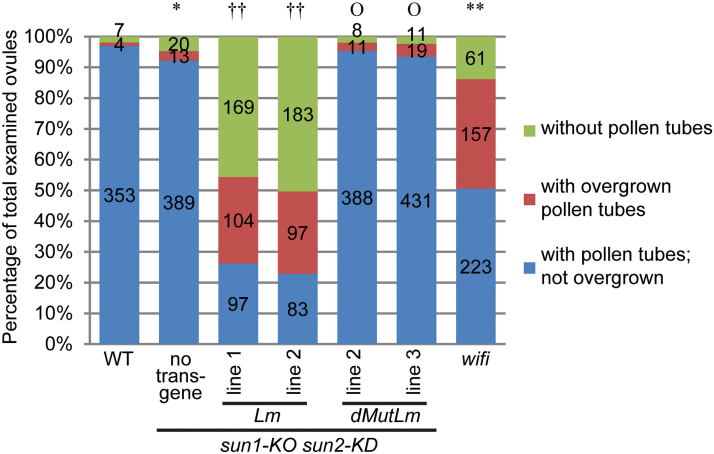
Quantification of pollen tube defects. Ovules with different pollen tube fates were quantified and shown in the stacked percentage column. The actual observed numbers are shown in each column. A single asterisk represents 0.05>*P*>0.01 when compared with the WT. Double daggers represent *P* <0.01 and ‘O’ represents *P* >0.05, when compared with *sun1-KO sun2-KD*. Double asterisks represent *P* <0.01 when compared with the WT. The Chi-square test was used.

To address whether the seed loss phenotype observed in Fig. 4 reflects defects in pollen tube reception, as described for WIP and WIT mutants, fertilized ovules were examined in *Lat52pro::ERS-RFP-SUN2Lm sun1-KO sun2-KD* or *Lat52pro::ERS-RFP-SUN2dMutLm sun1-KO sun2-KD* plants. When ovaries of *Lat52pro::ERS-RFP-SUN2Lm sun1-KO sun2-KD* were observed 3 d after the flowers opened, there were a small number of big ovules and a large number of small ovules (see Supplementary Fig. S5A at *JXB* online). The number of big ovules per ovary was similar to the seed number per silique, suggesting that the ovules that remain small at this stage do not develop into seeds (see Supplementary Fig. S5B at *JXB* online). Aniline blue staining showed that the big ovules were targeted by at least one pollen tube, suggesting that they were fertilized and developed into seeds. In small ovules, the central cell nucleus and the egg cell were visible (see Supplementary Fig. S5C at *JXB* online), suggesting that they were unfertilized. Among the total ovules examined, approximately one half (43% for line 1 and 52% for line 3) were without visible pollen tubes (Fig. 6; see Supplementary Table S1 at *JXB* online).

Ovaries of *sun1-KO sun2-KD* plants transformed with *Lat52pro::ERS-RFP-SUN2Lm* had randomly distributed big fertilized ovules (see Supplementary Fig. S5A at *JXB* online), and their pollen tubes were able to reach the receptacle end of a pistil (see Supplementary Fig. S6 at *JXB* online), suggesting that there is no defect in pollen tube growth and that, instead, the pollen tubes expressing ERS-RFP-SUN2Lm might have an ovular guidance defect (Higashiyama and Takeuchi, 2015). In addition, one-quarter of the ovules of *Lat52::ERS-RFP-SUN2Lm sun1-KO sun2-KD* examined bore overgrown pollen tubes (28% for line 1 and 25% for line 3, Fig. 6), a phenotype typical for a pollen tube reception defect. Both pollen tube defects were not observed in WT, *sun1-KO sun2-KD,* or *Lat52pro::ERS-RFP-SUN2dMutLm sun1-KO sun2-KD* (Fig. 6).

It has been shown previously that the pollen tubes of *wip123* and *wit12* have a high frequency of overgrowth in ovules and a minor defect in ovule targeting (Zhou and Meier, 2014). Since ERS-RFP-SUN2Lm can displace both WIP1 and WIT1 from the NE, the fate of *wifi* pollen tubes was characterized as a comparison. *wifi* pollen tubes are also able to reach the receptacle end of a pistil (see Supplementary Fig. S6 at *JXB* online). As shown in Fig. 6, and in Supplementary Table S1 at *JXB* online, approximately 14% of *wifi* ovules examined were without pollen tubes, which is less severe than the effect seen with *Lat52pro::ERS-RFP-SUN2Lm sun1-KO sun2-KD*. By contrast, approximately 36% of the *wifi* ovules examined had overgrown pollen tubes, which is more severe than that found for *Lat52pro::ERS-RFP-SUN2Lm sun1-KO sun2-KD*. Similar to *wip123* and *wit12* mutants, *wifi* also has a pronounced polytubey phenotype, which was not observed in the other mutants and transgenic lines including *Lat52pro::ERS-RFP-SUN2Lm sun1-KO sun2-KD,* suggesting that there are quantitative differences between the mutants examined.

## Discussion

### Function of SUN–WIP–WIT complexes

In vegetative tissues, SUNs, WIPs, and WITs form a complex at the NE that regulates nuclear shape and nuclear movement. Previously, it has been shown that WIPs and WITs at the pollen VNE are essential for VN migration (Zhou and Meier, 2014). In this study, ERS-RFP-SUN2Lm was specifically expressed in post-meiotic pollen grains and showed that it displaced GFP-WIP1 and GFP-WIT1 from the pollen VNE in the presence of both native SUN1 and SUN2 (Fig. 3), suggesting that ERS-RFP-SUN2Lm is able to outcompete KASH interactions with both SUN1 and SUN2. This is in line with our previous report that both SUN1 and SUN2 interact with WIP1, WIP2, and WIP3 (Zhou *et al.*, 2012). Expressing *ERS-RFP-SUN2Lm* in pollen grains of *sun1-KO sun2-KD* impaired the VN movement and caused severe seed loss, both of which were not observed in *sun1-KO sun2-KD* or *Lat52pro::ERS-RFP-SUN2dMutLm sun1-KO sun2-KD*. Furthermore, no seed loss was observed in *sun2-1*, supporting our preferred model where SUN1 and SUN2 redundantly anchor WIP and WIT at the VNE, and the SUN–WIP–WIT complex is responsible for VN migration during pollen tube growth. This suggests that WIP and WIT perform their role in male fertility in the context of a LINC complex and thus adds further evidence that the function of LINC complexes in nuclear migration is conserved in eukaryotes.

### Function of SUN, WIP, and WIT in pollen tube ovular guidance and reception

*sun1-KO sun2-KD* pollen grains expressing *ERS-RFP-SUN2Lm* have no defects in pollen viability and pollen tube growth. However, these pollen tubes have severe ovular guidance and reception defects, and similar phenotypes, yet with different severity, were observed in *wifi* (Fig. 6; see Supplementary Table S1 at *JXB* online). These severe phenotypes were not observed in *Lat52pro::ERS-RFP-SUN2dMutLm sun1-KO sun2KD* (Fig. 6; see Supplementary Table S1 at *JXB* online). Unlike *wifi*, *Lat52pro::ERS-RFP-SUN2Lm sun1-KO sun2KD* did not show an obvious polytubey phenotype. This can be explained by its strong ovular guidance defect, because of which not enough pollen tubes can target ovules to cause the polytubey phenotype. These data indicate that the SUN–WIP–WIT LINC complexes are involved in pollen tube ovular guidance and reception.

Pollen tube ovular guidance and reception involve signals from both female and male parts (Leydon *et al.*, 2014; for a recent review see Higashiyama and Takeuchi, 2015). Growing through pistil tissues, pollen tubes receive signals from female sporophytic tissues and undergo an activation or differentiation process which makes them responsive to attraction signals secreted from ovules (Leydon *et al.*, 2014; Higashiyama and Takeuchi, 2015). Known *Arabidopsis* genes expressed in pollen tubes that mediate ovular guidance include the ER-localized potassium transporters CHX21 and CHX23 (Lu *et al.*, 2011), the ER protein POD1 (Li *et al.*, 2011), membrane-anchored receptor-like cytoplasmic kinases LIP1 and LIP2 (Liu *et al.*, 2013), glycosylphosphatidylinositol-anchored protein COBL10 (Li *et al.*, 2013), glycosylphosphatidylinositol-biosynthesis-related proteins SETH1, SETH2, and APTG1(Lalanne *et al.*, 2004; Dai *et al.*, 2014), F-actin severing proteins MAP18 and MDP25 (Zhu *et al.*, 2013; Qin *et al.*, 2014), glutathione transferase GSTU26 (Lin *et al.*, 2014), xyloglucan endotransglucosylase/hydrolase XTH19 (Lin *et al.*, 2014), and mitogen-activated protein kinases MPK3 and MPK6 (Guan *et al.*, 2014). Three pollen-specific transcription factors MYB97, MYB101, and MYB120 have been reported to be essential for pollen tube reception (Leydon *et al.*, 2013; Liang *et al.*, 2013). Therefore, it is tempting to speculate that a transcriptionally-active VN at the growing pollen tube tip is required for locally expressing the male factors responsible for pollen tube guidance and reception during the late stages of pollen tube growth. As shown by our previous study, disrupting SUN–WIP–WIT LINC complexes can lead to a loss of the VN during pollen tube growth (Zhou and Meier, 2014), which would, by this model, lead to the pollen tube guidance and reception defects described here.

It was noted that *Lat52pro::ERS-RFP-SUN2dMutLm sun1-KO sun2KD* has a stronger ovular guidance defect than *wifi*. One possible explanation is that unidentified KASH proteins or other SUN-domain interacting proteins are involved in this specific aspect of male fertility and that they, too, are depleted in this mutant. Alternatively, the delocalized WIP and WIT proteins in pollen might cause this strong ovular guidance defect through unknown mechanisms.

### ERS-RFP-SUN2Lm as a tool to study the function of SUN proteins

ERS-RFP-SUN2Lm has been successfully used here to outcompete SUN1 and SUN2 for binding KASH proteins in mature pollen and pollen tubes and the function of SUN proteins in these two cell types has been revealed. SUN1 and SUN2 are expressed in various tissues (Graumann *et al.*, 2010; Oda and Fukuda, 2011) and probably play multiple roles in plant development. ERS-RFP-SUN2Lm can now be used as a tool to dissect the function of SUN proteins in a specific cell type or at a certain developmental stage without the interference of unrelated developmental phenotypes of a *sun* double null mutant.

## Supplementary data

Supplementary data can be found at *JXB* online.

Supplementary Fig. S1. ERS-GFP-SUN2Lm and ERS-GFP-SUN2dMutLm localize to the ER in *N. benthamiana*.

Supplementary Fig. S2. ERS-RFP-SUN2Lm delocalizes WIP1 and WIT1 from the NE in pollen grains.

Supplementary Fig. S3. Expression and localization pattern of SINE1 and SINE2 in WT pollen.

Supplementary Fig. S4. Both Lat52pro::ERS-RFP-SUN2Lm sun1-KO sun2-KD and Lat52pro::ERS-RFP-SUN2dMutLm *sun1-KO sun2-KD* transgenic *Arabidopsis* plants produce healthy pollen grains.

Supplementary Fig. S5. Fertilization defects in ovaries of *Lat52pro::ERS-RFP-SUN2Lm sun1-KO sun2-KD.*

Supplementary Fig. S6. Pollen tube growth in pistils revealed by aniline blue staining.

Supplementary Table S1. Pollen tube fate in ovaries 3 d after flower opening.

Supplementary Table S2. Primers used for cloning.

Supplementary Data
