# Supplementary material for: SUN anchors pollen WIP–WIT complexes at the vegetative nuclear envelope and is necessary for pollen tube targeting and fertility
Source: J Exp Bot. 2015 Sep 25;66(22):7299–307. doi: 10.1093/jxb/erv425 (PMC4765795; doi:10.1093/jxb/erv425)
Supplement: Supplementary Data [file supp_66_22_7299__index.html]

SUN anchors pollen WIP–WIT complexes at the vegetative nuclear envelope and is necessary for pollen tube targeting and fertility — SUN anchors pollen WIP–WIT complexes at the vegetative nuclear envelope and is necessary for pollen tube targeting and fertility — SUN anchors pollen WIP–WIT complexes at the vegetative nuclear envelope and is necessary for pollen tube targeting and fertility — Supplementary Data 

# SUN anchors pollen WIP–WIT complexes at the vegetative nuclear envelope and is necessary for pollen tube targeting and fertility

## Supplementary Data

Data files

- Supplementary Data - Supplementary Data
