# Supplementary material for: SUN anchors pollen WIP–WIT complexes at the vegetative nuclear envelope and is necessary for pollen tube targeting and fertility
Source: J Exp Bot. 2015 Sep 25;66(22):7299–307. doi: 10.1093/jxb/erv425 (PMC4765795; doi:10.1093/jxb/erv425)
Supplement: Supplementary Data [file supp_erv425_jexbot154997_file001.pdf]

SUN anchors pollen WIP-WIT complexes at the vegetative nuclear envelope and is necessary for pollen tube targeting and fertility

Xiao Zhou, Norman Reid Groves, and Iris Meier

Supplementary Data

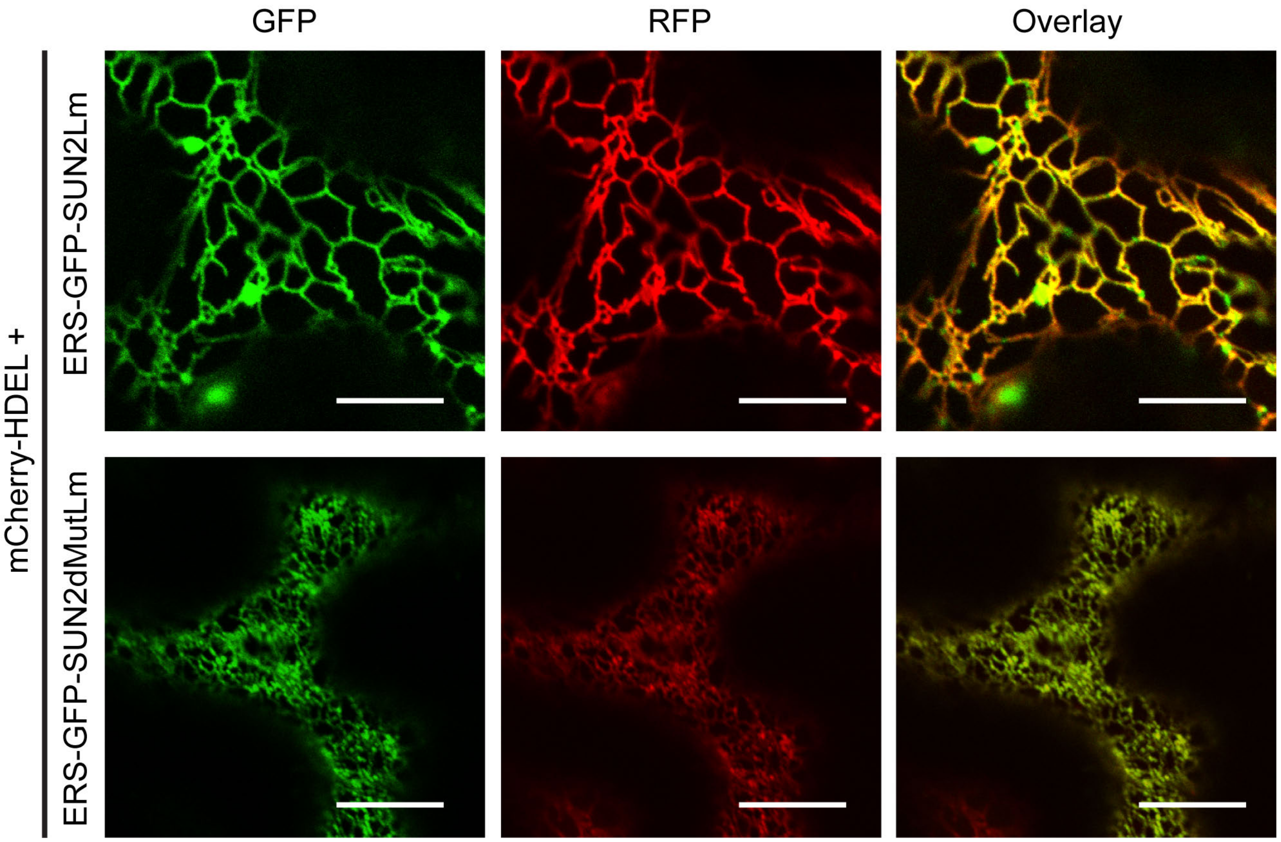

Figure S1. ERS-GFP-SUN2Lm and ERS-GFP-SUN2dMutLm localize to the ER in *N. benthamiana*.

An mCherry-ER marker was co-expressed with *35Spro::ERS-GFP-SUN2Lm* and *35Spro::ERS-GFP-SUN2dMutLm* in *N. benthamiana*, respectively. Both ERS-GFP-SUN2Lm and ERS-GFP-SUN2dMutLm were colocalized with mCherry-ER at the ER network. Scale bar equals 10µm.

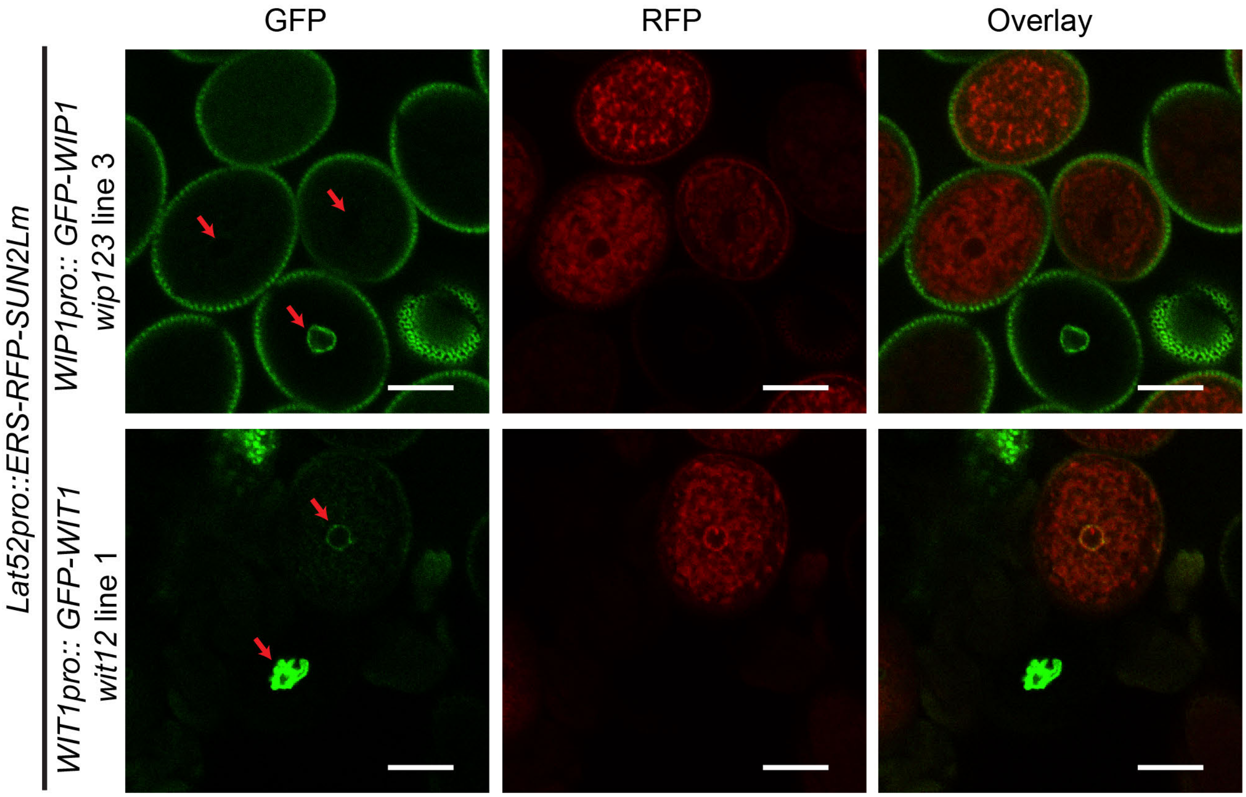

Figure S2. ERS-RFP-SUN2Lm delocalizes WIP1 and WIT1 from the NE in pollen grains.
ERS-RFP-SUN2Lm driven by *Lat52pro* was expressed in the pollen of *WIP1pro::GFP-WIP1 wip123* and *WIT1pro::GFP-WIT1 wit12*, respectively. The pollen grains of heterozygous plants were examined. The GFP channel was adjusted to resolve the VNE localization (red arrows in the GFP channel images) of GFP-WIP1 or GFP-WIT1. Some pollen grains are without visible VN because the VN were not in the confocal imaging plane. Scale bars =10µm.

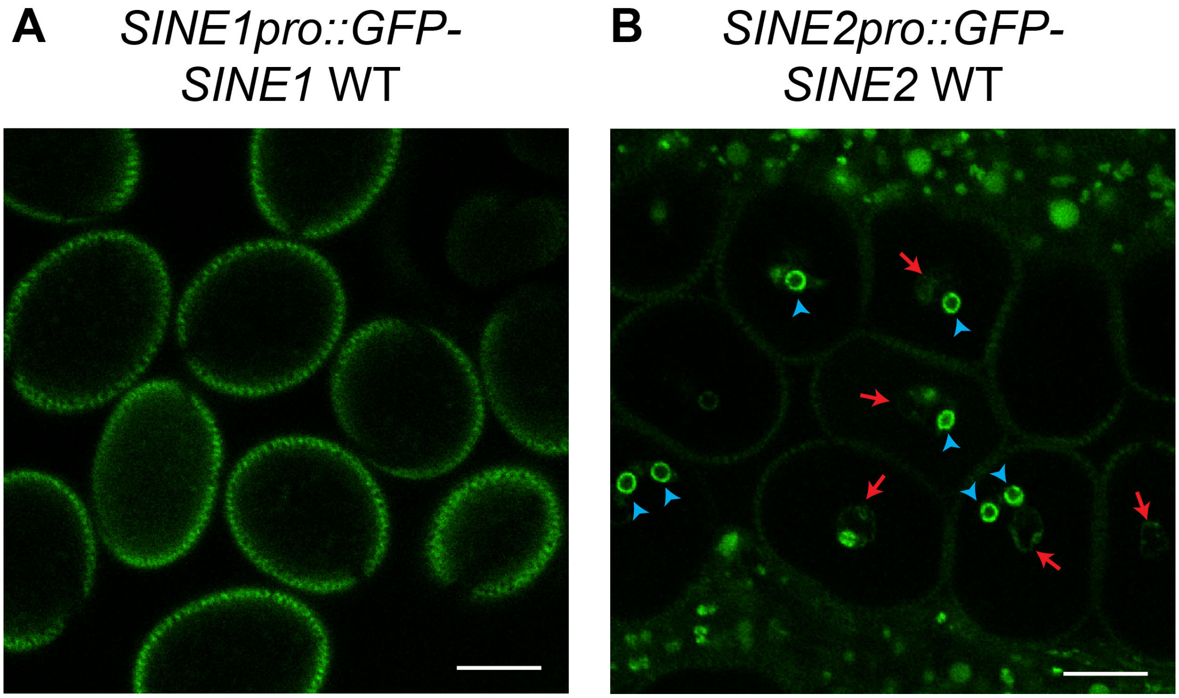

Figure S3. Expression and localization pattern of SINE1 and SINE2 in WT pollen.

(A) *GFP-SINE1* driven by *SINE1 promoter* (*SINE1pro::GFP-SINE1*) shows no expression in pollen. (B) *GFP-SINE2* driven by *SINE2 promoter* (*SINE2pro::GFP-SINE2*) is localized at the VNE (red arrows) and the SC NE (blue arrowheads). The GFP-SINE1 image was obtained at a higher gain setting than the GFP-SINE2 image, and therefore shows stronger autofluorescence of the pollen cell wall. Scale bars =10 µm.

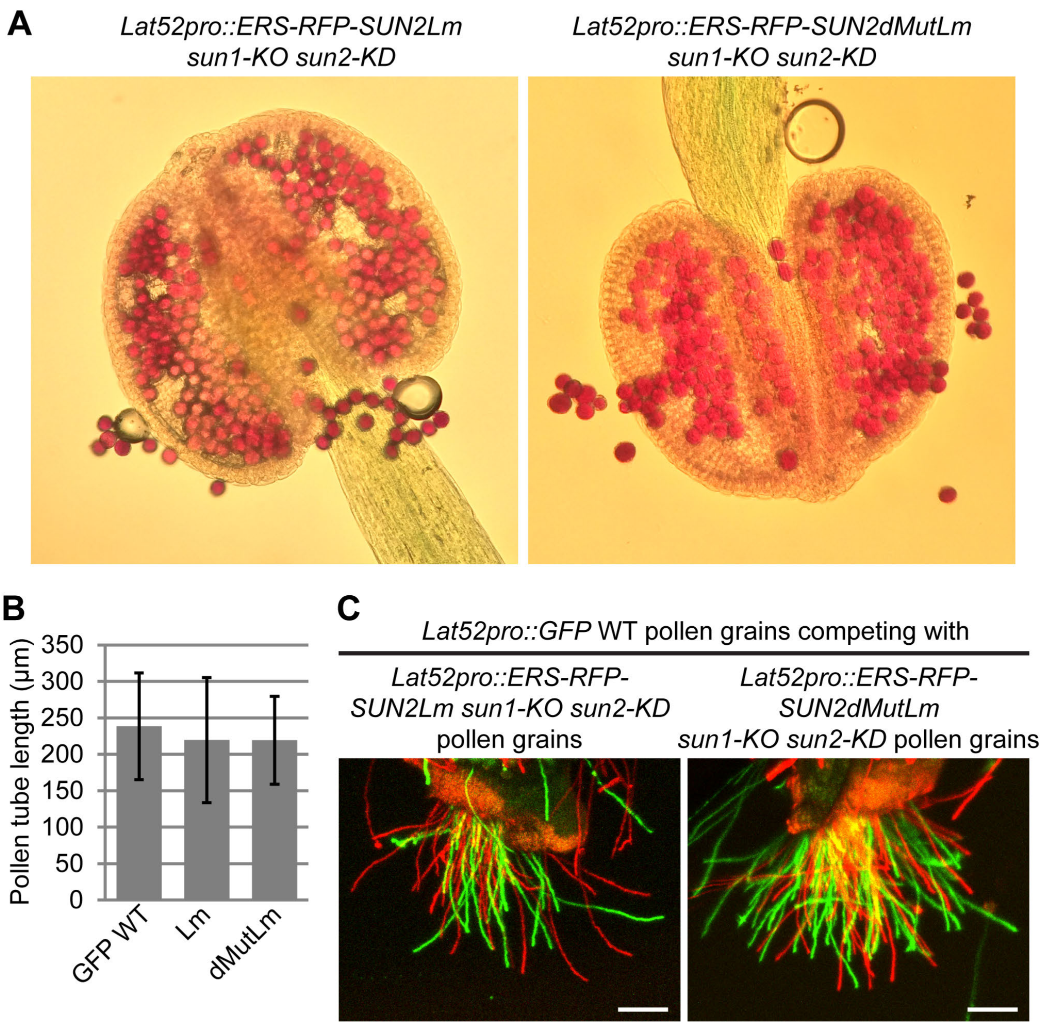

Figure S4. Both Lat52pro::ERS-RFP-SUN2Lm sun1-KO sun2-KD and Lat52pro::ERS-RFP-SUN2dMutLm sun1-KO sun2-KD transgenic Arabidopsis plants produce healthy pollen grains.
(A) Pollen grains were stained using Alexander's staining. No obvious dead pollen grains were found.
(B) Pollen tube length was measured after 5h *in vitro* pollen germination. "Lm" represents *Lat52pro::ERS-RFP-SUN2Lm sun1-KO sun2-KD* and "dMutLm" represents *Lat52pro::ERS-RFP-SUN2dMutLm sun1-KO sun2-KD*. No significant difference was found among samples. One-way analysis of variance (α < 0.01) followed by Tukey's honest significant difference test (α < 0.01) was used.
(D) No overall pollen germination defects and pollen tube growth defects were observed when *Lat52pro::ERS-RFP-SUN2Lm sun1-KO sun2-KD* pollen or *Lat52pro::ERS-RFP-SUN2dMutLm sun1-KO sun2-KD* pollen competed with *Lat52pro::GFP* WT pollen on a WT stigma. One-half of a stage 14 WT stigma was saturated with *Lat52pro::GFP wit12* pollen, and the other one-half was saturated with the tested pollen. Pollen tubes were imaged 5 h later using confocal microscopy. Each image is a maximum projection of a z-stack image. Scale bars = 100 µm.

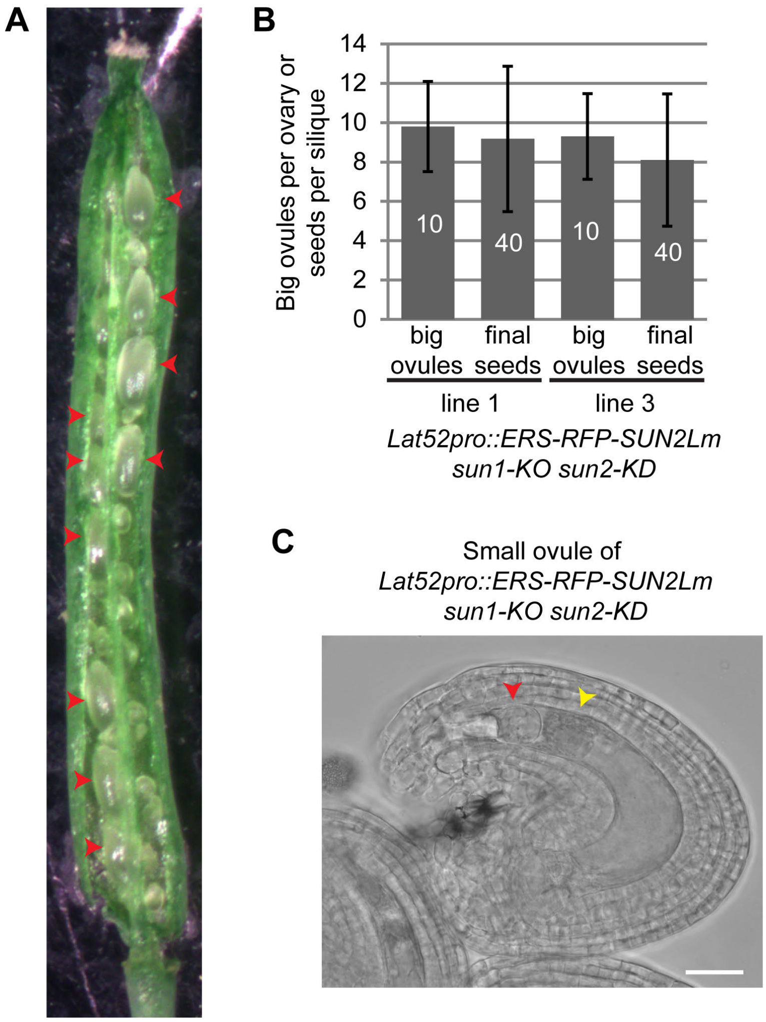

Figure S5. Fertilization defects in ovaries of *Lat52pro::ERS-RFP-SUN2Lm sun1-KO sun2-KD*

(A) A small number of big ovules (red arrowhead) and a large number of small ovules were observed in ovaries of *Lat52pro::ERS-RFP-SUN2Lm sun1-KO sun2-KD*.

(B) The number of big ovules per ovary and the number of seeds per silique in *Lat52pro::ERS-RFP-SUN2Lm sun1-KO sun2-KD* were compared. No significant difference was found among samples. One-way analysis of variance ($\alpha < 0.01$) followed by Tukey's honest significant difference test ($\alpha < 0.01$) was used. N of each sample is indicated in the histogram.

(C) The central cell nucleus (yellow arrowhead) and the egg cell (red arrowhead) can be observed in the small ovule of *Lat52pro::ERS-RFP-SUN2Lm sun1-KO sun2-KD*. Scale bar equal 50 µm.

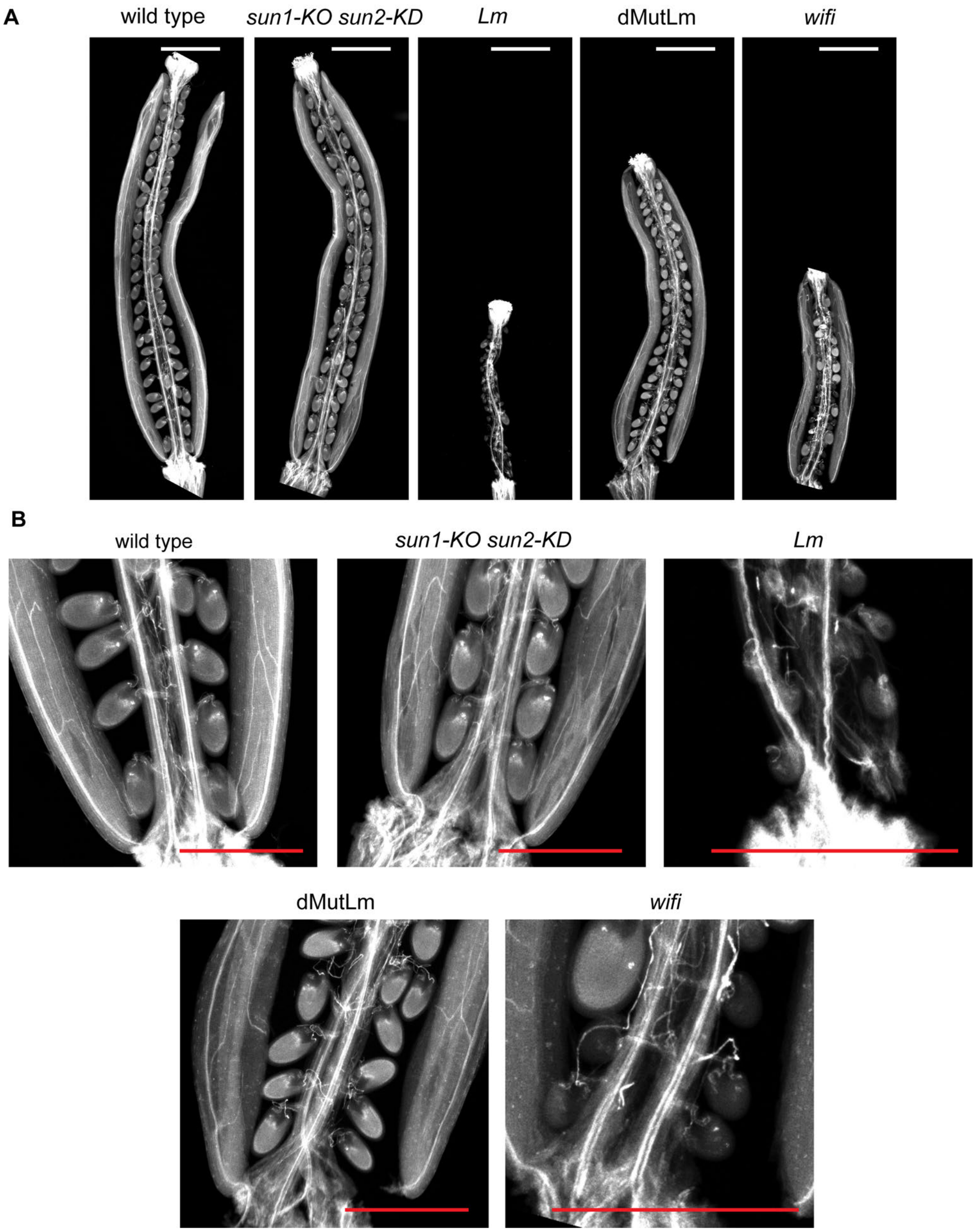

Figure S6. Pollen tube growth in pistils revealed by aniline blue staining

Overview of pollen tubes in pistils 3 days after flower opening are shown in (A) and pollen tubes at the receptacle end were enlarged and shown in (B). “Lm” represents *Lat52pro::ERS-RFP-SUN2Lm sun1-KO sun2-KD*. “dMutLm” represents *Lat52pro::ERS-RFP-SUN2dMutLm sun1-KO sun2-KD*. Scale bars = 500 µm.

Table S1 Pollen tube fate in ovaries 3 days after flower opening

	WT			*sun1-KO sun2-KD*			Lm line 1			Lm line 3			dMutLm line 2			dMutLm line 3			*wifi*		
	Big fertilized ovules	Small unfertilized ovules	Row Marginal Total	Big fertilized ovules	Small unfertilized ovules	Row Marginal Total	Big fertilized ovules	Small unfertilized ovules	Row Marginal Total	Big fertilized ovules	Small unfertilized ovules	Row Marginal Total	Big fertilized ovules	Small unfertilized ovules	Row Marginal Total	Big fertilized ovules	Small unfertilized ovules	Row Marginal Total	Big fertilized ovules	Small unfertilized ovules	Row Marginal Total
With pollen tubes; not overgrown	348	5	353	378	11	389	71	26	97	49	34	83	384	4	388	426	5	431	164	59	223
With overgrown pollen tubes	3	1	4	8	5	13	2	102	104	4	93	97	9	2	11	10	9	19	17	140	157
Without pollen tubes	0	7	7	0	20	20	0	169	169	0	183	183	0	8	8	0	11	11	0	61	61
Column Marginal Total	351	13	364	386	36	422	73	297	370	53	310	363	393	14	407	436	25	461	181	260	441
Polytubey among Column Marginal Total	7	0	7	14	1	15	4	2	6	2	3	5	17	0	17	16	1	17	15	37	52

"Lm" represents *Lat52pro::ERS-RFP-SUN2Lm sun1-KO sun2-KD*. "dMutLm" represents *Lat52pro::ERS-RFP-SUN2dMutLm sun1-KO sun2-KD*.

Table S2. Primers used for cloning

Primer Name	Sequence
Lat52proinF	CTCAAGCTAAGCTTGAGCTCGACATACTCGACTCAGAAGGTATTG
Lat52proinR	GGGCAATGATGCTTGTGAGTCTGGCCATTTTTTTTTTGGTGTGTGTAC
2SAlbuminERF	ATGGCCAGACTCACAAGCATCATTGCCCTCTTCGCAGTGGCTCTGCT GGTTGCAGATGCGTACGCCTACCGCACCACTAGTATGGCCTCCT
2SAlbminERinR	AGGAGGCCATACTAGTGGTG
SUN2LmF	CACCATGAGGAAATTGACTTTGAAGGATT
SUN2LmR	TCACAATTCATCATGGTGGTGATGGTGATGCCCACCTCCAGCATGAG CAACAGAGACTGA
35SERSinF	AGGCGGCCGCACTAGTATGGCCAGACTCACAAGCAT
35SERSinR	TGCTCACCATACTAGTGGTGCGGTAGGCGTACGCAT
